# Lipid metabolism and m^6^A RNA methylation are altered in lambs supplemented rumen-protected methionine and lysine in a low-protein diet

**DOI:** 10.1186/s40104-022-00733-z

**Published:** 2022-07-13

**Authors:** Kefyalew Gebeyew, Chao Yang, Hui Mi, Yan Cheng, Tianxi Zhang, Fan Hu, Qiongxian Yan, Zhixiong He, Shaoxun Tang, Zhiliang Tan

**Affiliations:** 1 CAS Key Laboratory for Agro-Ecological Processes in Subtropical Region, National Engineering Laboratory for Pollution Control and Waste Utilization in Livestock and Poultry Production, Hunan Provincial Key Laboratory of Animal Nutritional Physiology and Metabolic Process, Institute of Subtropical Agriculture, The Chinese Academy of Sciences, Changsha, 410125 Hunan China; 2 University of Chinese Academy of Science, Beijing, 100049 China; 3 Hunan Co-Innovation Center of Animal Production Safety, CICAPS, Changsha, 410128 Hunan China

**Keywords:** Lambs, Lipid metabolism, Low-protein, Lysine, Methionine, m^6^A RNA methylation

## Abstract

**Background:**

Methionine or lysine has been reported to influence DNA methylation and fat metabolism, but their combined effects in N6-methyl-adenosine (m^6^A) RNA methylation remain unclarified. The combined effects of rumen-protected methionine and lysine (RML) in a low-protein (LP) diet on lipid metabolism, m^6^A RNA methylation, and fatty acid (FA) profiles in the liver and muscle of lambs were investigated. Sixty-three male lambs were divided into three treatment groups, three pens per group and seven lambs per pen. The lambs were fed a 14.5% crude protein (CP) diet (adequate protein [NP]), 12.5% CP diet (LP), and a LP diet plus RML (LP + RML) for 60 d.

**Results:**

The results showed that the addition of RML in a LP diet tended to lower the concentrations of plasma leptin (*P* = 0.07), triglyceride (*P* = 0.05), and non-esterified FA (*P* = 0.08). Feeding a LP diet increased the enzyme activity or mRNA expression of lipogenic enzymes and decreased lipolytic enzymes compared with the NP diet. This effect was reversed by supplementation of RML with a LP diet. The inclusion of RML in a LP diet affected the polyunsaturated fatty acids (PUFA), n-3 PUFA, and n-6 PUFA in the liver but not in the muscle, which might be linked with altered expression of FA desaturase-1 (*FADS1*) and acetyl-CoA carboxylase (*ACC*). A LP diet supplemented with RML increased (*P* < 0.05) total m^6^A levels in the liver and muscle and were accompanied by decreased expression of fat mass and obesity-associated protein (*FTO*) and alkB homologue 5 (*ALKBH5*). The mRNA expressions of methyltransferase-like 3 (*METTL3*) and methyltransferase-like 14 (*METTL14*) in the LP + RML diet group were lower than those in the other two groups. Supplementation of RML with a LP diet affected only liver YTH domain family (*YTHDF2*) proteins (*P* < 0.05) and muscle *YTHDF3* (*P* = 0.09), which can be explained by limited m^6^A-binding proteins that were mediated in mRNA fate.

**Conclusions:**

Our findings showed that the inclusion of RML in a LP diet could alter fat deposition through modulations of lipogenesis and lipolysis in the liver and muscle. These changes in fat metabolism may be associated with the modification of m^6^A RNA methylation.

**Graphical abstract:**

A systematic graph illustrates the mechanism of dietary methionine and lysine influence on lipid metabolism and M^6^A. The green arrow with triangular heads indicates as activation and brown-wine arrows with flat heads indicates as suppression. 
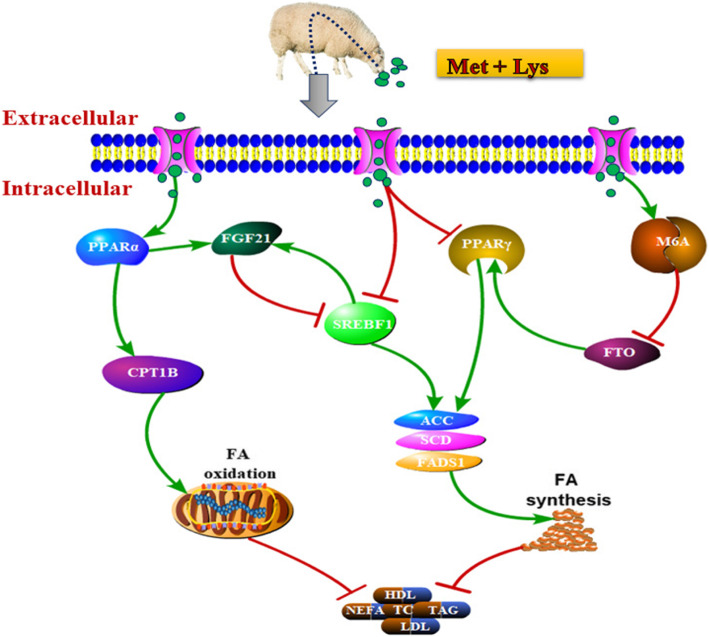

**Supplementary Information:**

The online version contains supplementary material available at 10.1186/s40104-022-00733-z.

## Introduction

Increased price of dietary protein sources and environmental concerns leading to feeding trends towards reduced dietary protein levels to meat-producing animals, including bulls and pigs [1, 2]. Reducing the levels of dietary protein below the NRC recommendation is the most successful nutritional approach in lowering feed cost and nitrogen emissions [3]. However, feeding a low-protein (LP) diet has been shown to affect the carcass characteristics by reducing the carcass lean content and increasing fat accretion depending on the levels of dietary protein reduction and its amino acids composition [2, 4]. It also increased fat deposition by increasing the quantity of energy accessible for fat mass and restricting protein synthesis [5, 6]. One possible explanation could be a deficiency of one or more essential amino acid (EAA). Adding dietary EAA to the LP diet may balance the fat accretion and improve protein synthesis. In this regard, dietary methionine (Met) and lysine (Lys), top-limiting EAA in growing lambs, have been reported to promote protein synthesis and modulate lipid metabolism [7, 8].

A diet containing deficient or adequate Met levels affected lipid turnover by altering the expression of genes-related to fat metabolism in the liver and other tissues [8, 9]. The inclusion of low Met levels in the LP diet has little influence on fat accumulation, but those in a high protein diet reduced fat accretion [5], suggesting that the effects of Met in the LP diet are influenced by other EAAs in the diet. Meanwhile, increasing the levels of dietary Lys has been reported to reduce lipid accretion and improve muscle protein synthesis through regulating key genes involved in various pathways [7]. This effect was connected with improved biosynthesis of L-carnitine, which is reported to decrease carcass fat content and liver total lipids [10, 11]. Conversely, feeding Lys deficient diet increased intramuscular fat content in *longissimus dorsi* muscle [12]. The findings stated in the above studies point out that dose-dependent influences of dietary Met or Lys in lipid metabolism. However, the combined effects of Met and Lys on the mechanisms of controlling fat removal and accretion and de novo fatty acid synthesis in liver and muscle tissues of lambs under LP diet conditions are not clarified so far. In addition, considering the essence of evaluating N6-methyl-adenosine (m^6^A) methylation in the liver and muscle and the vital role of liver tissue in lipoprotein and lipid metabolism, the present study focused on the liver and muscle tissues even though the main site of de novo FAs synthesis in sheep is adipose tissue.

Methionine and betaine serve as donors of methyl groups that are involved in the DNA and m^6^A RNA methylation process. In this content, supplementation of dietary Met affected the DNA methylation process by altering the SAM concentration [13]. Likewise, supplementation of dietary betaine affected the process of m^6^A RNA methylation [14, 15]. These researches suggested a modulatory role of methyl donors in epigenetic modification. The m^6^A is considered as the most common form of intrinsic messenger RNA modification in eukaryotic cells [16]. It is a dynamic and reversible process, and installation of a methyl group to m^6^A is catalyzed by methyltransferases like 3 (METTL3), methyltransferases like 14 (METTL14), and Wilms’ tumor 1-associating protein (WTAP) [17]. Fat mass and obesity-associated (FTO) and alkB homologue 5 (ALKBH5) have been identified as demethylase [18, 19], which promote the dumping of a methyl group from m^6^A via different mechanisms. Subsequently, the YTH domain family (YTHF1–3) proteins have recognized the modification of m^6^A to influence mRNA maturation, splicing stability, decay, translation, and export [16]. These modifications of RNA could affect several biological functions, such as lipid metabolism and energy homeostasis [20]. Moreover, alters in the m^6^A RNA methylation and lipid metabolism in various tissues may be inter-related mechanisms in response to supplementation of Met and Lys. Thus, we hypothesized that the inclusion of rumen protected methionine and lysine (RML) in the LP diet may influence lipid metabolism through modulations of m^6^A RNA methylation in growing lambs. Therefore, the study aimed to investigate m^6^A RNA methylation, lipid metabolism, and fatty acid profiles in the liver and *longissimus dorsi* muscle of lambs fed a LP diet supplemented with RML.

## Materials and methods

Animal Care and Use Committee of the Institute of Subtropical Agriculture were checked and approved all the experimental protocols used in this trial (202020). Animals were handled in accordance with the protocol approved by the committee. The feeding trial was performed in Hulunbuir city, Inner Mongolia Autonomous Region, China.

### Animals, diets, and experimental design

This experimental procedure was in conjunction with our earlier research [21]. Briefly, the rumen-protected lysine (RPL, LysiPEARL) and methionine (RPM, MetiPEARL) products were obtained from Kemin Industries Inc. (Iowa, USA). The RPL and RPM products are contained L-Lys monohydrochloride at 475 g/kg and DL-Met at 500 g/kg, respectively. The rumen passage rates of Lys and Met were about 85% and 66%, respectively, [22]. At four months of age and the initial body weight (BW) of 24.82 ± 2.03 kg, sixty-three fat tail male Hulunbuir lambs were randomly allocated into three dietary groups with three pens per group and seven lambs per pen. The lambs were fed a 14.5% crude protein (CP) diet (positive control, adequate-protein diet [NP]); a 12.5% CP diet (negative control; LP); and a LP diet with RML (LP + RML). The quantity of RPM (0.253 g/kg DM) and RPL (0.50 g/kg DM) supplemented to the lambs were selected following our previous results [23, 24].

The total experimental period was 75 d, including 15 d of acclimatization and 60 d of actual periods. The compositions and nutrient levels of the experimental diets are shown in Tables 1 and 2. Determination of dry matter (DM), ash, and crude protein (CP) of the feed were carried out using the standard protocol of the Association of Official Analytical Chemists (AOAC) [25]. Acid-detergent fiber (ADF) and neutral detergent fiber (NDF) were measured following the methods of Van Soest et al. [26]. The isothermal automatic calorimeter (5E-AC 8018, Changsha Kaiyuan Instruments Co. Ltd., Changsha, China) was used to assay the gross energy (GE) content of feed. Extraction of total amino acids (AA) from the experimental feed was carried out according to the standard procedure descried by the AOAC [27]. The quantification of individual amino acids from the extract was performed using HPLC (Agilent 1100; Agilent Technologies) following the standard protocol of Henderson et al. [28].

**Table 1:** Ingredients and chemical composition (% DM basis) of the treatment diets

Item	Treatment^a^		
	NP	LP	LP + RML
Ingredient, % of DM basis			
Corn	20.00	26.0	26.00
Barley	20.87	20.87	20.87
Wheat bran	8.72	8.72	8.72
Soybean meal	12.5	6.5	6.5
Cottonseed meal	3.0	3.0	3.0
Sodium bicarbonate	0.7	0.7	0.7
Salt	0.5	0.5	0.5
Magnesium oxide	0.5	0.5	0.5
Calcium carbonate	0.2	0.2	0.2
Beet molasses	1.0	1.0	1.0
Wheat straw	30.0	30.0	30.0
Premix^b^	2.0	2.0	2.0
Total	100	100	100
Rumen protected methionine, g/kg of DM	–	–	0.253
Rumen protected lysine, g/kg of DM	–	–	0.5
Chemical composition, % of DM			
DM	95.1	95.1	94.8
Ash	10.2	10.4	10.3
CP	14.57	12.46	12.46
NDF	34.1	33.2	30.5
ADF	15.2	14.1	13.3
Metabolize energy, MJ/kg	9.55	9.55	9.55
GE, MJ/kg	17.05	17.04	17.01

^a^*NP* adequate protein diet, *LP* low protein diet, *LP + RML* low protein diet with rumen-protected methionine and lysine

^b^Supplied per kilogram of dietary DM: 221.31 mg of Fe, 16.23 mg of Cu, 71.31 mg of Zn, 71.31 mg of Mn, 1.23 mg of I, 0.54 mg of Co, 1.70 mg of Se, 16,250 IU of vitamin A, 5000 IU of vitamin D, and 40 IU of vitamin E

**Table 2:** Amino acids and fatty acid composition of the experimental diets

Item	Treatment^a^		
	NP	LP	LP + RML
Amino acid composition, %			
Asp	0.88	0.85	0.84
Ser	0.46	0.43	0.44
Glu	1.82	1.56	1.55
Gly	0.53	0.43	0.45
Ala	0.88	0.84	0.85
Pro	0.87	0.75	0.73
Thr	0.44	0.42	0.43
Val	0.65	0.62	0.61
Met	0.25	0.18	0.58
Ile	0.43	0.39	0.40
Leu	1.07	0.90	0.91
Tyr	0.34	0.29	0.31
Phe	0.57	0.53	0.52
Lys	0.83	0.75	1.69
His	0.39	0.31	0.31
Arg	0.60	0.57	0.58
Fatty acids composition, %			
C14:0	0.69	0.68	0.67
C16:0	18.51	19.29	19.44
C18:0	10.41	10.93	11.02
C18:1n-9c	17.01	15.81	15.54
C18:2n-6c	45.90	45.97	46.01
C18:3n-3	7.49	7.31	7.32

^a^*NP* adequate protein diet, *LP* low protein diet, *LP + RML* low protein diet with rumen-protected methionine and lysine

### Sample collection

Blood was sampled on d 60 (before morning feeding) from the jugular vein of lambs into 5-mL heparinized tubes (Changsha Yiqun Medical Equipment Co., Ltd., Hunan, China) to analyze plasma hormone and lipid-related metabolites. The blood plasma was separated by centrifugation at 3000 × *g* for 15 min at room temperature and frozen at − 80 °C until analysis. Seven or six lambs per treatment with BW close to the group weight were chosen for slaughter. After evisceration, the weight of the carcass was recorded within 30 min of slaughter. The liver samples were taken quickly, sealed in foil paper, and stored at − 80 °C for later analysis. Then, the *longissimus dorsi* muscle (muscle) was sampled at the 12/13th rib of each carcass, sealed in foil paper, and stored at − 80 °C until analysis. The dressing percentage was computed as described by Wang et al. [4].

### Analysis of plasma metabolite and hormones

The concentrations of plasma triglycerides (TAG), high-density lipoprotein-cholesterol (HDL-C), total cholesterol (TC), and low-density lipoprotein-cholesterol (LDL-C) were analyzed using a Cobas C311 analyzer (Roche Di-agnostics, Rotkreuz, Switzerland) based on the procedure described by Wang et al. [4]. The non-esterified fatty acid (NEFA), leptin (LEP), adiponectin (ADPN), and insulin (INSU) concentrations were assayed using the enzyme-linked immunosorbent assay (ELISA) kits specific to sheep following the protocol given by the manufacturer’s manual (Fankel Co., Ltd., Shanghai, China) as described by Gebeyew et al. [29]. The results are presented as pg/mL. The sensitivity of the kits was 0.1 pg/mL.

### Measurement of tissue enzyme activity, concentration of S-adenosylhomocysteine and L-carnitine

Liver and muscle samples were weighed (100 mg) and homogenized (1:9 w/v) with ice-cold normal saline buffer. Then, the upper liquids were separated by centrifugation at 3000 × *g* for 15 min at 4 °C to detect enzyme activity. Another 100 mg of liver and muscle samples were weighed and homogenized (1:9 w/v) using a mixture of n-butanol, methanol, and deionized water at the ratio of 5:25:70 (v:v:v). Then, the supernatants were retained by centrifugation at 5000 × *g* for 10 min at 4 °C to detect the L-carnitine. The activities of fibroblast growth factor 21 (FGF21), hormone-sensitive lipase (HSL), acetyl-CoA carboxylase (ACC), malic enzyme (ME), lipoprotein lipase (LPL), carnitine palmitoyltransferase I B (CPT1B), fatty acid-binding protein 4 (FABP4), fatty acid desaturase 1 (FADS1), and stearoyl-CoA desaturase (SCD) were determined in the liver and muscle tissues using the ELISA kits specific to sheep following the recommended procedure (Jiangsu Meimian industrial Co., Ltd., Yancheng, China). The results were presented as pg/mg protein or ng/mg protein. The intra-assay and inter-assay coefficient of variation (CV) of all ELISA kits used to detect FGF21, HSL, ACC, LPL, CPT1B, FABP4, FADS1, SCD, and ME were < 10% and < 12%, respectively. The protein concentrations in the liver and muscle tissues were quantified using the BCA protein assay kit (Beyotime Biotech Inc., Shanghai, China). The levels of S-adenosylhomocysteine (SAH) and L-carnitine were measured using the ELISA kits specific to sheep based on the manufacturers’ protocol (Fankewei Co., Ltd., Shanghai, China). The results are presented as ng/g or pg/g [30]. The intra-assay and inter-assay CV of ELISA kits used to SAH and L-Carnitine were < 8% and < 10%, respectively.

### Fatty acid profiling

The liver and muscle tissue samples were freeze-dried. Then, the total lipids from lyophilized samples were extracted using the chloroform-methanol (2:1, v/v) following the methods described elsewhere [31]. Fatty acid methyl esters (FAME) of the tissues and experimental diets were prepared using a solution of KOH-methanol as reported by Tan et al. [32] . The FAME was identified by an Agilent 7890A gas chromatographer (GC) system equipped with a flame ionization detector (Agilent Technologies Inc., Santa Clara, CA, USA) as previously described by Wang et al. [4]. The concentration of each fatty acids was quantified by comparing the retention times of the peak area with those authentic standards (Sigma Chemical Co., St. Louis, MO, USA), and presented as a percentage of total fatty acids. The partial sums of saturated fatty acids (SFA), monounsaturated fatty acids (MUFA), polyunsaturated fatty acids (PUFA), n-3 PUFA, and n-6 PUFA were computed.

### RNA extraction and gene expression profiling

Cytoplasmic RNA was isolated from liver and muscle tissues of lambs using the cytoplasmic and nuclear RNA purification kit following the kit procedures (Norgen Biotek Corp., Thorold, ON, Canada). The concentration and purity of the extracted RNA were checked using a NanoDrop 2000 spectrophotometer (Thermo Fisher Scientific, Waltham, MA, USA). The integrity of total RNA was verified by agarose-formaldehyde (1%) gel electrophoresis. Aliquots of RNA samples were reverse transcribed into complementary DNA (cDNA) using Evo M-MLV Reverse Transcription Kit (AG11707, Accurate Biotechnology Co., Ltd., Changsha, Hunan, China) following the manufacturer’s guidelines. Then, the synthesized cDNA was placed at − 20 °C until analysis.

The lipid metabolism-related genes investigated in this study were *ACC*, *SCD*, *HSL*, *LPL*, *CPT1B*, *FABP4*, *FGF21*, adipose triglyceride lipase (*ATGL*), fatty acid synthase (*FASN*), sterol regulatory element-binding protein 1 (*SREBF1*), peroxisome proliferator-activated receptor-gamma (*PPARγ*), and peroxisome proliferator-activated receptor-alpha (*PPARα*) (Additional file 1: Table S1). As internal reference genes, expression of ribosomal protein S9 (*RPS9*), *β-actin*, and glyceraldehyde-3-phosphate dehydrogenase (*GAPDH*) were analyzed. The stability of the reference genes were assessed using BestKeeper, Genorm, and NormFinder tools [33]. The *RPS9* and *GAPDH* were identified as the most stable reference gene across experimental treatment. Therefore, the two reference genes were used to normalize the data. The amplification efficiency of all primers used in this study was computed using serial cDNA dilution curves. All primers showed efficiency between 90% and 110%, and correlation coefficients were close to 1.0. All gene-specific primers sequences used in the present study were synthesized by Sangon Biotech Co., Ltd. (Shanghai, China). The real-time PCR was performed using SYBR® Green Premix *Pro Taq* HS qPCR Kit (AG11707, Accurate Biology Co., Ltd., Changsha, China) on a fluorescence LightCycler 480 II system (Roche, Basel, Switzerland) following the manufacturer’s direction. The details of the reaction program were described elsewhere [34]. The relative mRNA expressions of the target genes were calculated using the 2^-ΔΔCt^ method [35].

### Quantification of m^6^A content

The m^6^A RNA methylation quantification kit (Epigentek Group Inc., Farmingdale, NY, USA) was used to detect the total content of m^6^A in liver and muscle tissues according to the protocol given by the manufacturer’s manual. The total content of m^6^A was computed using the formula: m^**6**^A % = {[(OD_Sample_ – OD_NC_)/S] ÷ [(OD_PC_ – OD_NC_)]} × 100%, and S, NC, and PC denote the total quantity of input RNA, negative control and positive control, respectively, as described by Heng et al. [17].

### Statistical analysis

Statistical analyses were executed using origin pro 2020b and the SPSS version 23.0 (SPSS Inc., Chicago, IL, USA). First, Shapiro–Wilk and Levene’s tests were employed to confirm the normality and homoscedasticity of data, respectively. Then, Tukey’s post hoc tests were used to determine the differences between treatment means, significant difference was regards as *P* < 0.05 and trends were recognized 0.05 ≤ *P* < 0.1. The data are presented as the means ± standard errors of the mean (SEMs). The correlations between genes-related to lipid metabolism and methylation were performed using Spearman’s rank correlation coefficient in R package and the network was constructed on the Omics-studio (LC-Bio Technology Co., Ltd., Hangzhou, China).

## Results

### Carcass parameter, lipid-related metabolites, and hormones

**Fig. 1:**
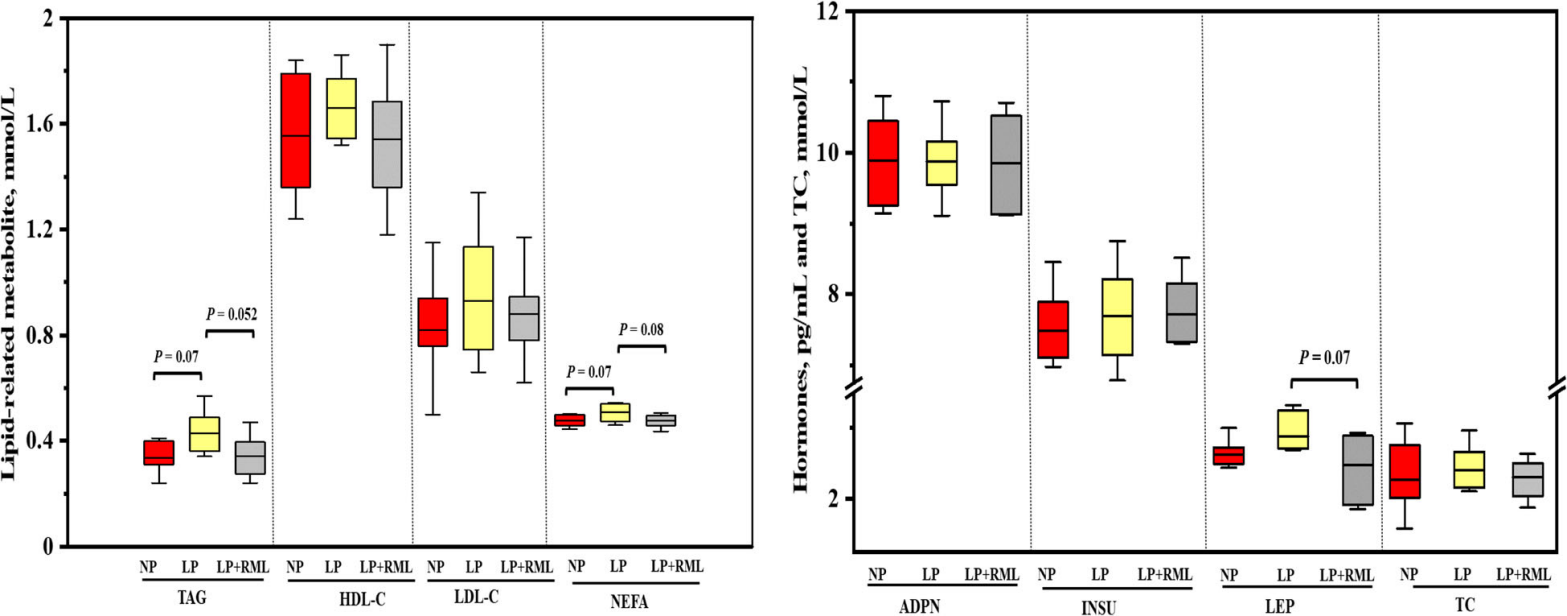
Effect of a low-protein diet supplemented with rumen-protected methionine and lysine on lipid-related metabolites and hormone levels. Values are expressed as means ± SEM indicated by vertical bars. Different letters indicate a significant difference among three dietary treatments (*P* < 0.05). NP, adequate protein diet; LP, low-protein diet; LP + RML, low-protein diet supplemented with rumen-protected methionine and lysine

**Fig. 2:**
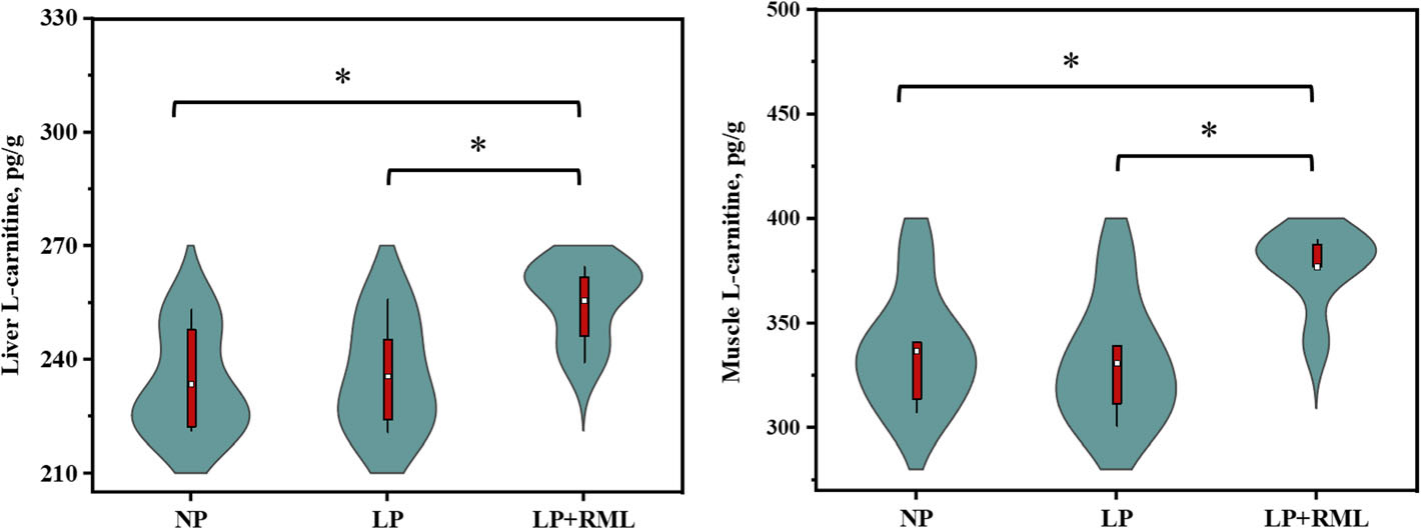
Effect of a low-protein diet supplemented with rumen-protected methionine and lysine on L-carnitine levels in liver and muscle. On each side of the gray line is a kernel density estimation to illustrate the distribution of shape of the data in a group. Wider and skinnier sections of the violin plot represent a higher and lower probability. The white dot represents the mean and the thick red bar in the center represents the interquartile range. * = significantly different means (*P* < 0.05). NP, adequate protein diet (*n* = 7); LP, low-protein diet (*n* = 6); LP + RML, low-protein diet supplemented with rumen-protected methionine and lysine (*n* = 7)

The live body weight, average daily gain and feed intake, G:F ratio, dressing percentage, and hot carcass weight were similar (*P* > 0.05) among the three dietary groups (Table 3). The concentrations of plasma TAG and NEFA tended to be increased in the LP diet group than the other two groups (*P* < 0.1, Fig. 1). The concentration of plasma LEP (*P* = 0.07) in the LP + RML diet group tended to be lower than that in the LP diet group. In comparison to the other two groups, the concentrations of L-carnitine in the liver and muscle were enhanced (*P* < 0.05) in the RML supplemented group (Fig. 2).

**Table 3:** Effect of a low-protein diet supplemented with rumen-protected methionine and lysine on live body weight, hot carcass weight, and dressing percentage

Items	Treatments			SEM	*P*-value
	NP	LP	LP + RML		
LBW, kg	36.22	36.44	36.91	0.774	0.826
ADG, kg/d	0.195	0.187	0.195	0.017	0.940
ADFI, kg/d	1.458	1.418	1.436	0.014	0.163
G:F	0.134	0.132	0.136	0.012	0.977
HCW, kg	17.04	17.58	18.22	0.359	0.102
DP, %	47.50	48.49	49.44	1.641	0.737

Data are presented as means with their pooled SEM

*NP* adequate protein diet, *LP* low-protein diet, *LP + RML* low-protein diet supplemented with rumen-protected methionine and lysine, *LBW* Live body weight, kg, *ADG* Average daily gain, kg/d, *ADFI* Average daily feed intake, kg/d; G:F: ADG/ADFI, *HCW* hot carcass weight, kg, *DP* dressing percentage, %

### Fatty acids profiles in liver and muscle tissues

The compositions of FA in the liver and muscle tissues are presented in Tables 4 and 5. In liver, the C16:0 concentration was greater (*P* < 0.05) in the LP diet group than that in the NP diet group, but comparable with the RML supplemented group. The concentration of C16:1 was greater (*P* < 0.05) in the LP and LP + RML diet groups than that in the NP diet group. In comparison to the NP diet group, the concentration of C18:2n-6c was reduced in the LP diet group but equivalent to the LP + RML diet group. The LP diet group had a higher (*P* < 0.05) concentration of C18:1n-9 t than those in the other two groups. In comparison to the NP diet group, the LP and LP + RML diet groups had lower (*P* < 0.05) concentrations of C18:3n-3, C20:3n-6, n-3 and n-6 PUFA. The LP diet group had greater (*P* < 0.05) SFA and lower (*P* < 0.05) PUFA and n-6 PUFA than that in the NP diet group but were equivalent to the RML supplemented group. However, neither a LP diet nor LP + RML affected (*P* > 0.05) the partial sums of MUFA in the liver. In muscle, the concentrations of C15:0 and C18:3n-3 in the LP and LP + RML diet groups were reduced (*P* < 0.05) than that of the NP diet group. In addition, the n-3 PUFA concentration tended to be reduced (*P* = 0.07) in the LP and LP + RML diet groups. Conversely, the LP diet increased (*P* < 0.05) the concentrations of C18:1n-9 t and C20:4n-6 than that of the other two diets. The partial sums of SFA, PUFA, and n-6 PUFA in the muscle were unaffected (*P* > 0.05) by dietary groups.

**Table 4:** Effect of a low-protein diet supplemented with rumen-protected methionine and lysine on the fatty acids composition of liver in growing lambs, % of total fatty acids

Fatty acids, %	Treatment			SEM	*P*-value
	NP	LP	LP + RML		
C12:0	0.239	0.244	0.256	0.012	0.563
C14:0	2.625	2.862	2.885	0.128	0.318
C14:1	0.169	0.168	0.164	0.021	0.883
C15:0	0.277	0.329	0.316	0.015	0.062
C16:0	23.84^b^	26.08^a^	24.86^ab^	0.561	0.040
C16:1	1.552^b^	2.086^a^	2.119^a^	0.138	0.020
C17:0	0.992	1.049	1.014	0.075	0.875
C18:0	15.649	15.21	15.28	0.502	0.035
C18:1n-9 t	0.358^b^	0.423^a^	0.356^b^	0.015	0.011
C18:1n-9c	41.619	40.608	40.989	0.548	0.441
C18:2n-6c	8.496^a^	7.204^b^	7.787^ab^	0.288	0.029
C20:0	0.159	0.158	0.173	0.018	0.861
C18:3n-3	2.465^a^	2.173^b^	2.194^b^	0.071	0.019
C20:3n-6	0.323^a^	0.233^b^	0.231^b^	0.019	0.005
C20:4n-6	0.325	0.296	0.305	0.032	0.861
C24:0	0.544	0.587	0.606	0.051	0.699
C22:6n-3	0.368	0.297	0.362	0.043	0.533
Fatty acid partial sums					
SFA^1^	44.32^b^	46.52^a^	45.40^ab^	0.433	0.009
MUFA^2^	43.69	43.28	43.63	0.551	0.721
PUFA^3^	11.98^a^	10.20^b^	10.97^ab^	0.308	0.007
∑n-6 PUFA^4^	9.14^a^	7.73^b^	8.41^ab^	0.276	0.018
∑n-3 PUFA^5^	2.83^a^	2.47^b^	2.56^b^	0.069	0.005

Different letters across a row indicate a significant difference among the three dietary treatments (*P* < 0.05). Data are presented as means with their pooled SEM

*NP* adequate protein diet (*n* = 7), *LP* low-protein diet (*n* = 6), *LP + RML* low-protein diet supplemented with rumen-protected methionine and lysine (*n* = 7)

^1^SFA (saturated fatty acid) = C12:0 + C14:0 + C15:0 + C16:0 + C17:0 + C18:0 + C20:0 + C24:0

^2^MUFA (monounsaturated fatty acid) = C14:1 + C16:1 + C18:1n-9 t + C18:1n-9c

^3^PUFA (polyunsaturated fatty acid) = C18:2n-6c + C18:3n-3 + C20:3n-6 + C20:4n-6 + C22:6n-3

^4^n-3 PUFA = C18:3n-3 + C22:6n-3

^5^n-6 PUFA = C18:2n-6c + C20:3n-6 + C20:4n-6

**Table 5:** Effect of a LP diet supplemented with rumen-protected methionine and lysine on the fatty acids composition of muscle in growing lambs, % of total fatty acids

Fatty acids, %	Treatment			SEM	*P*-value
	NP	LP	LP + RML		
C12:0	0.627	0.769	0.482	0.132	0.174
C14:0	2.895	3.080	3.003	0.167	0.754
C14:1	0.227	0.217	0.235	0.014	0.890
C15:0	0.981^a^	0.678^b^	0.715^b^	0.037	< 0.001
C16:0	23.95	24.72	25.01	0.641	0.503
C16:1	2.222	2.234	2.210	0.115	0.988
C17:0	1.182	0.976	0.923	0.087	0.288
C18:0	18.03	17.90	17.24	0.859	0.794
C18:1n-9 t	0.636^b^	0.750^a^	0.501^b^	0.045	0.006
C18:1n-9c	39.06	39.11	39.65	0.616	0.759
C18:2n-6c	5.988	5.450	5.860	0.216	0.256
C20:0	0.318	0.293	0.259	0.034	0.470
C18:3n-3	2.369^a^	2.206^b^	2.209^b^	0.041	0.018
C20:3n-6	0.260	0.247	0.286	0.024	0.515
C20:4n-6	0.301^b^	0.448^a^	0.306^b^	0.023	< 0.001
C24:0	0.330	0.347	0.369	0.016	0.231
C22:6n-3	0.619	0.578	0.594	0.080	0.868
Fatty acid partial sums					
SFA^1^	48.31	48.76	48.00	0.751	0.769
MUFA^2^	42.15	42.31	42.59	0.644	0.883
PUFA^3^	9.538	8.930	9.400	0.259	0.353
∑n-6 PUFA^4^	6.550	6.145	6.595	0.244	0.466
∑n-3 PUFA^5^	2.988	2.785	2.804	0.062	0.066

Different letters across a row indicate a significant difference among the three dietary treatments (*P* < 0.05). Data are presented as means with their pooled SEM. *NP*, adequate protein diet (*n* = 7); *LP*, low-protein diet (*n* = 6); *LP + RML*, low-protein diet supplemented with rumen-protected methionine and lysine (*n* = 7)

^1^SFA (saturated fatty acid) = C12:0 + C14:0 + C15:0 + C16:0 + C17:0 + C18:0 + C20:0 + C24:0

^2^MUFA (monounsaturated fatty acid) = C14:1 + C16:1 + C18:1n-9 t + C18:1n-9c

^3^PUFA (polyunsaturated fatty acid) = C18:2n-6c + C18:3n-3 + C20:3n-6 + C20:4n-6 + C22:6n-3

^4^n-3 PUFA = C18:3n-3 + C22:6n-3

^5^n-6 PUFA = C18:2n-6c + C20:3n-6 + C20:4n-6

### Enzyme activity in the liver and muscle tissues

As depicted in Table 6, adding RML in a LP diet depressed (*P* < 0.05) ACC, ME, and SCD enzyme activities in the liver compared with the LP diet. However, those enzyme activities were unaffected in muscle (*P* > 0.05) by the supplementation of RML with a LP diet except for SCD (Table 7). In liver and muscle, the CPT1B enzyme activity in the LP + RML diet group was higher (*P* < 0.05) than that in the other two diet groups. In comparison with the LP diet group, the LP + RML diet group increased (*P* < 0.05) the activity of HSL only in the liver. The LP diet increased (*P* < 0.05) the enzyme activities of FABP4 and LPL in the liver and muscle compared with the NP and LP + RML diets. Meanwhile, the LP diet increased (*P* < 0.05) the FADS1 and FGF-21 enzyme activities only in the liver. However, no variations were found between dietary groups for FADS1, FGF21, and HSL enzyme activities in the muscle (*P* > 0.05).

**Table 6:** Effect of a low-protein diet supplemented with rumen-protected methionine and lysine on liver tissue enzyme activities

Items	Treatments			SEM	*P*-value
	NP	LP	LP + RML		
ACC, ng/mg protein	9.77^b^	12.52^a^	8.59^b^	0.229	< 0.001
CPT1B, ng/mg protein	30.89^b^	30.03^b^	42.80^a^	2.564	0.007
FABP4, pg/mg protein	244.13^b^	295.78^a^	253.96^b^	12.742	0.015
FADS1, pg/mg protein	14.66^b^	17.81^a^	13.62^b^	0.372	< 0.001
FGF21, pg/mg protein	90.51^b^	110.84^a^	111.62^a^	5.326	0.020
HSL, ng/mg protein	0.234^b^	0.226^b^	0.282^a^	0.009	0.002
LPL, ng/mg protein	9.91^b^	13.56^a^	9.76^b^	0.530	< 0.001
ME, ng/mg protein	3.89^b^	4.82^a^	3.74^b^	0.308	0.008
SCD, ng/mg protein	0.343^b^	0.497^a^	0.313^b^	0.063	0.010

Different letters across a row indicate a significant difference among the three dietary treatments (*P* < 0.05). Data are presented as means with their pooled SEM

*NP* adequate protein diet (*n* = 7), *LP* low-protein diet (*n* = 6), *LP + RML* low-protein diet supplemented with rumen-protected methionine and lysine (*n* = 7)

**Table 7:** Effect of a low-protein diet supplemented with rumen-protected methionine and lysine on enzyme activities in muscle tissue

Items	Treatments			SEM	*P*-value
	NP	LP	LP + RML		
ACC, ng/mg protein	9.59	9.61	9.23	0.80	0.934
CPT1B, ng/mg protein	31.39^b^	30.57^b^	43.36^a^	2.77	0.012
FABP4, pg/mg protein	176.89^b^	220.87^a^	176.08^b^	8.53	0.004
FADS1, pg/mg protein	14.70	15.32	14.52	0.57	0.603
FGF21, pg/mg protein	88.36	97.62	91.82	3.96	0.296
HSL, ng/mg protein	0.240	0.252	0.262	0.02	0.616
LPL, ng/mg protein	9.71^b^	11.45^a^	9.48^b^	0.43	0.010
ME, ng/mg protein	3.83	4.20	3.76	0.18	0.236
SCD, ng/mg protein	0.34^a^	0.35^a^	0.26^b^	0.02	0.016

Different letters across a row indicate a significant difference among the three dietary treatments (*P* < 0.05). Data are presented as means with their pooled SEM

*NP* adequate protein diet (*n* = 7), *LP* low-protein diet (*n* = 6), *LP + RML* low-protein diet supplemented with rumen-protected methionine and lysine (*n* = 7)

### Lipid metabolism related genes in the liver and muscle tissues

**Fig. 3:**
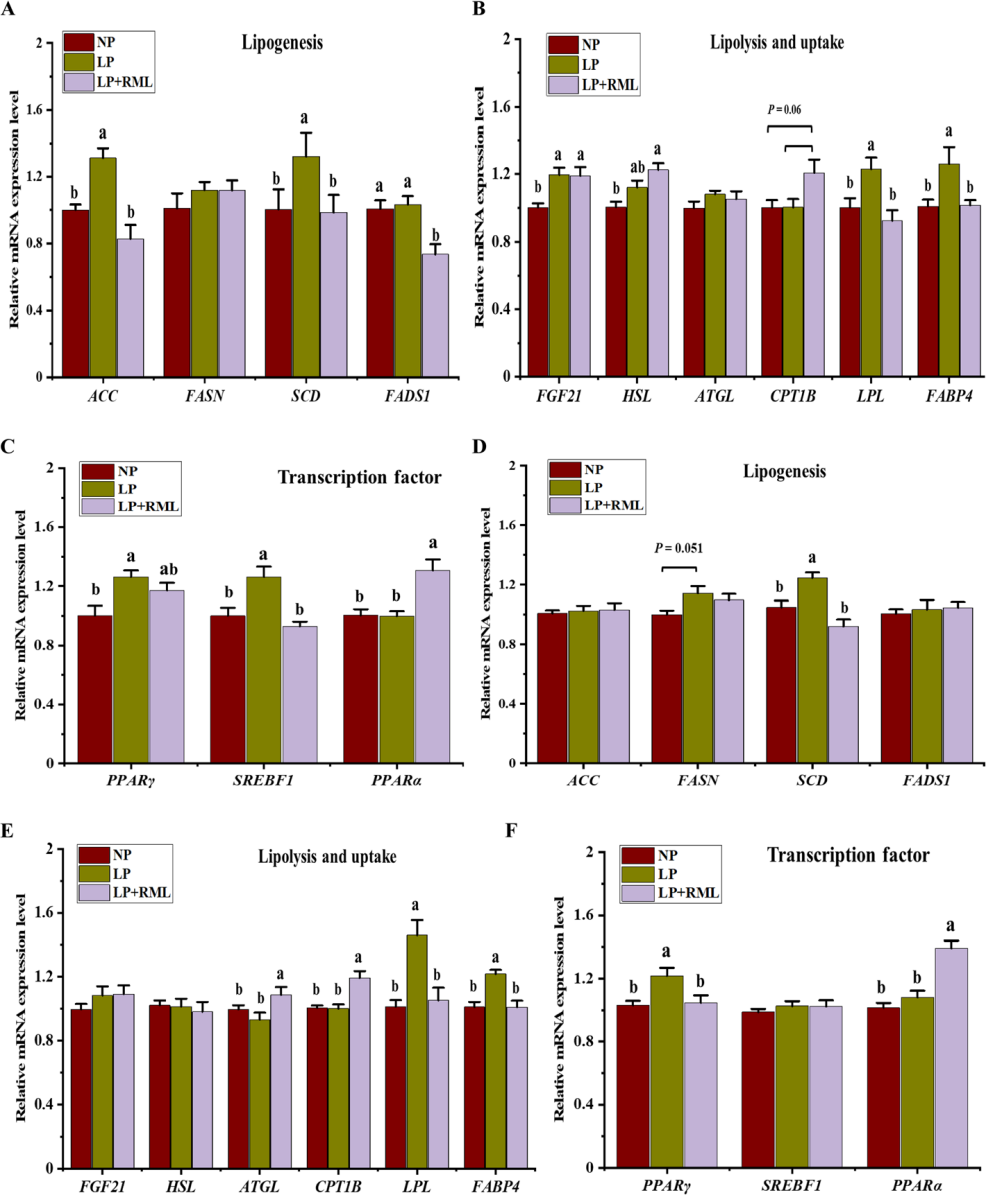
Effect of a low-protein diet supplemented with rumen-protected methionine and lysine on the expression of genes involved in fat metabolism in the liver (**A**, **B**, **C**) and muscle (**D**, **E**, **F**). Data are expressed as means ± SEM indicated by vertical bars. Different letters indicate a significant difference among the three dietary treatments (*P* < 0.05). NP, adequate protein diet (*n* = 7); LP, low-protein diet (*n* = 6); LP + RML, low-protein diet supplemented with rumen-protected methionine and lysine (*n* = 7)

In the liver, the mRNA expressions of *ACC*, *SCD*, *LPL*, *FABP4*, and *SREBF1* were greater (*P* < 0.05) in the LP diet group than those in the other two groups (Fig. 3). Meanwhile, the expression of *FADS1* (*P* < 0.05) was down-regulated in the LP + RML diet group compared with the other two groups (Fig. 3A). Conversely, the expression of *CPT1B* tended to be enhanced (*P* = 0.06) in the LP + RML diet group than those in the other two groups (Fig. 3B). In comparison to the NP diet group, the mRNA expression of *FGF21* was up-regulated (*P* < 0.05) in the LP and LP + RML diet groups. The mRNA expressions of *HSL* and *PPARα* were up-regulated (*P* < 0.05) by the supplementation of RML with a LP diet, while mRNA expression of *PPARγ* in the LP + RML diet group was similar (*P* > 0.05) with the other two groups (Fig. 3C). However, the mRNA expressions of liver *FASN* and *ATGL* were unaffected (*P* > 0.05) by dietary treatments. In muscle, the mRNA expressions of *SCD*, *LPL*, *FABP4*, and *PPARγ* were up-regulated (*P* < 0.05) in the LP diet group than those in the other two groups (Fig. 3D-F). In addition, the mRNA expression of *FASN* tended to be up-regulated (*P* = 0.051) in the LP diet group than that of in the NP diet group. Furthermore, the mRNA expressions of *CPT1B*, *ATGL*, and *PPARα* were elevated (*P* < 0.05) in the LP + RML diet group than those in the other two dietary groups. However, the mRNA expressions of *ACC*, *FADS1*, *HSL*, *SREBF1*, and *FGF21* in the muscle were similar (*P* > 0.05) among the three dietary groups.

### The m^6^A RNA methylation and its related gene expression in liver and muscle tissues

**Fig. 4:**
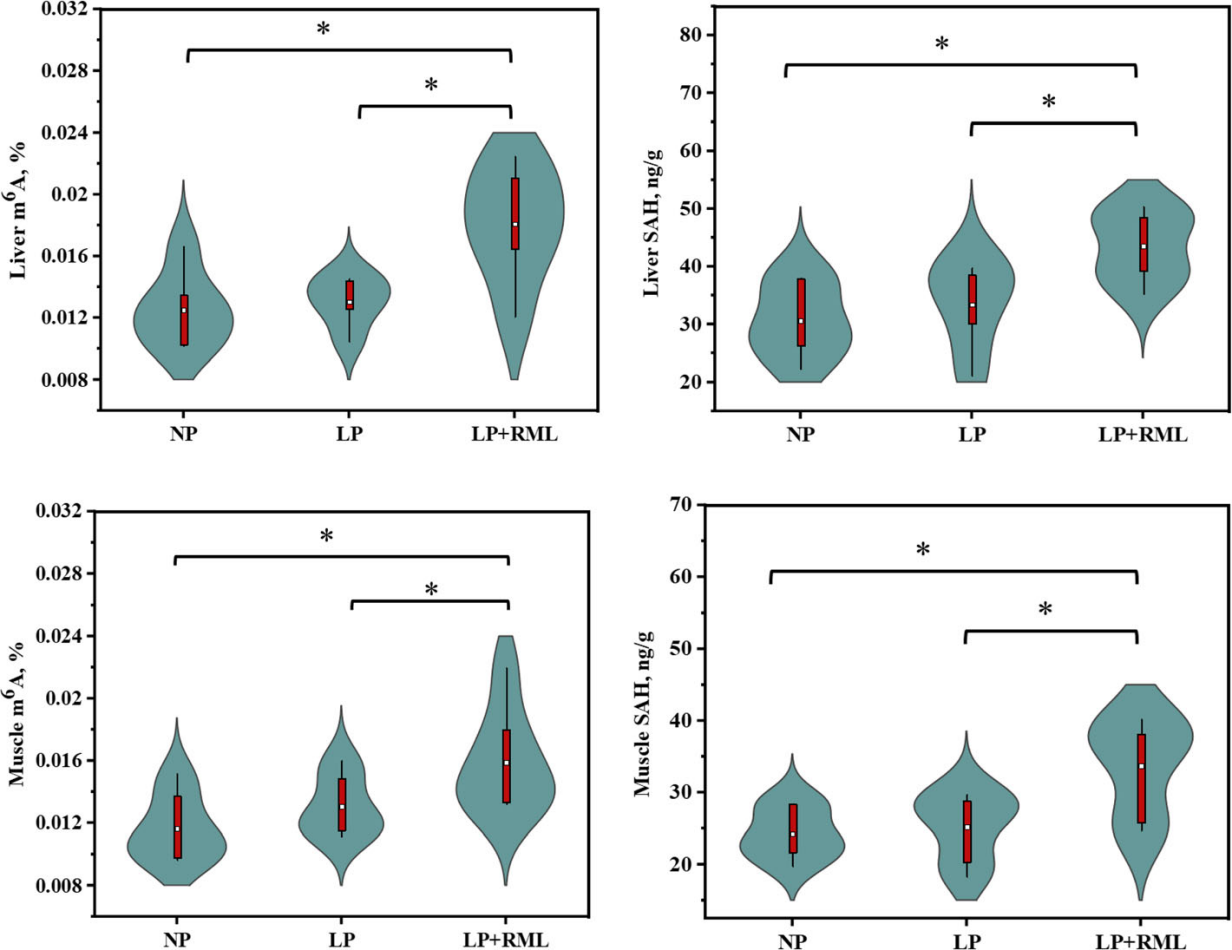
Effect of a low-protein diet supplemented with rumen-protected methionine and lysine on the m^6^A RNA methylation and SAH levels in liver and muscle. On each side of the gray line is a kernel density estimation to illustrate the distribution of shape of the data in a group. Wider and skinnier sections of the violin plot represent a higher and lower probability. The white dot represents the mean and the thick red bar in the center represents the interquartile range. * = significantly different means (*P* < 0.05). NP, adequate protein diet (*n* = 7); LP, low-protein diet (*n* = 6); LP + RML, low-protein diet supplemented with rumen-protected methionine and lysine (*n* = 7)

**Fig. 5:**
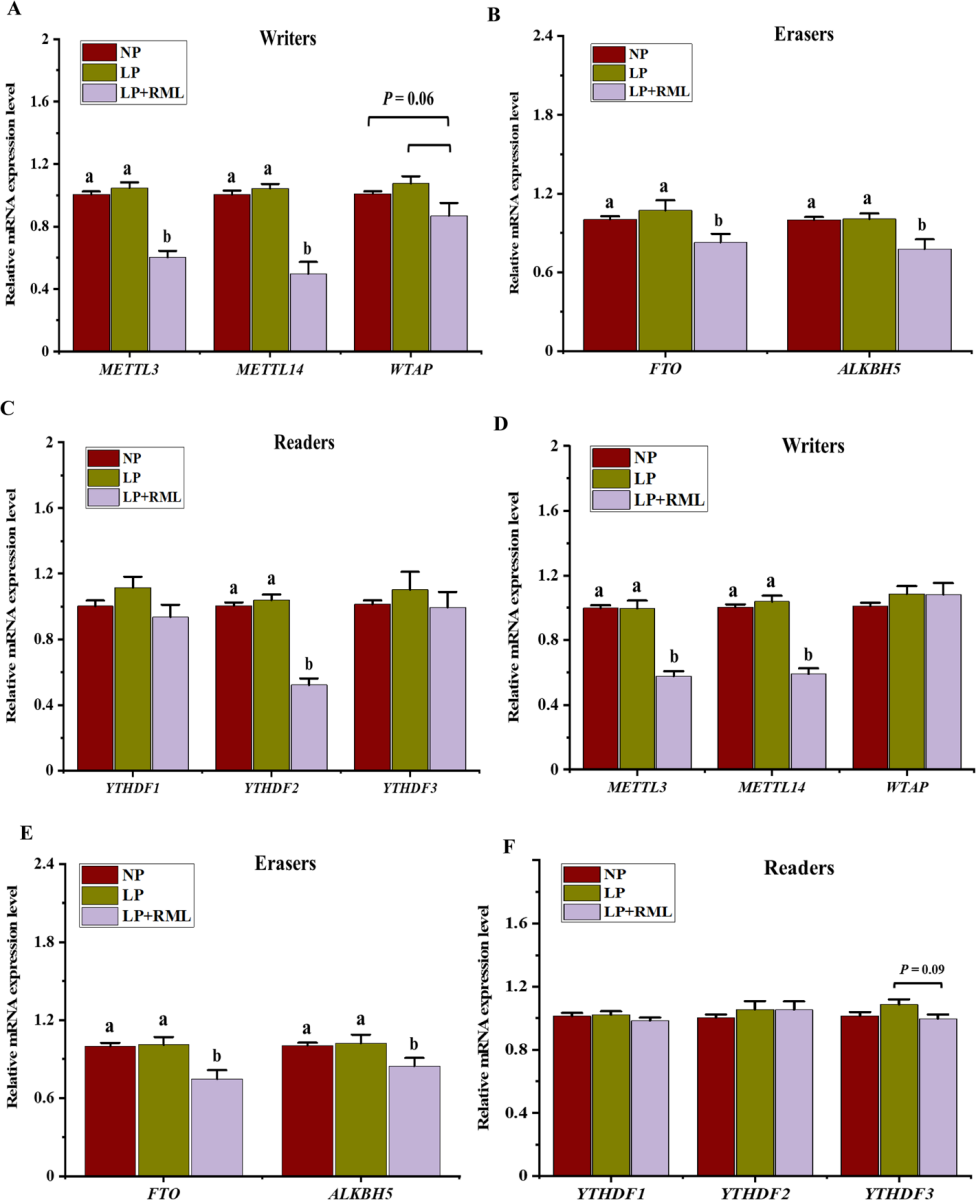
Effect of a low-protein diet supplemented with rumen-protected methionine and lysine on the expression of genes related to m^6^A RNA methylation in liver (**A**, **B**, **C**) and muscle (**D**, **E**, **F**). Data are expressed as means ± SEM indicated by vertical bars. Different letters indicate a significant difference among the three dietary treatments (*P* < 0.05). NP: adequate protein diet (*n* = 7), LP: low-protein diet (*n* = 6), LP + RML: low-protein diet supplemented with rumen-protected methionine and lysine (*n* = 7)

The total content of m^6^A and SAH and relative mRNA expressions were performed to reveal the impacts of an LP diet supplemented with and without RML on the mechanism of regulating m^6^A RNA methylation in the liver and muscle tissues. The addition of RML in a LP diet improved (*P* < 0.05) the total content of m^6^A and SAH in the liver and muscle (Fig. 4). In liver, a LP diet supplemented with RML down-regulated (*P* < 0.05) the mRNA expression levels of *METTL3/14*, *ALKBH5*, *FTO*, and *YTHDF2* (Fig. 5A-C). Likewise, mRNA expression levels of *METTL3/14*, *WTAP*, *FTO*, and *ALKBH5* in the muscle were down-regulated (*P* < 0.05) by the supplementation of RML with a LP diet (Fig. 5D-F). In addition, the mRNA expression levels of *WTAP* (*P* = 0.06) in the liver and *YTHD3* (*P* = 0.09) in the muscle tended to be depleted in the LP + RML diet group, whereas the gene expressions of muscle *WTAP*, *YTHDF1* and *YTHDF2* were similar (*P* > 0.05) among the three groups.

### Correlations among genes-associated to m^6^A RNA methylation and lipid metabolism in the liver and muscle

**Fig. 6:**
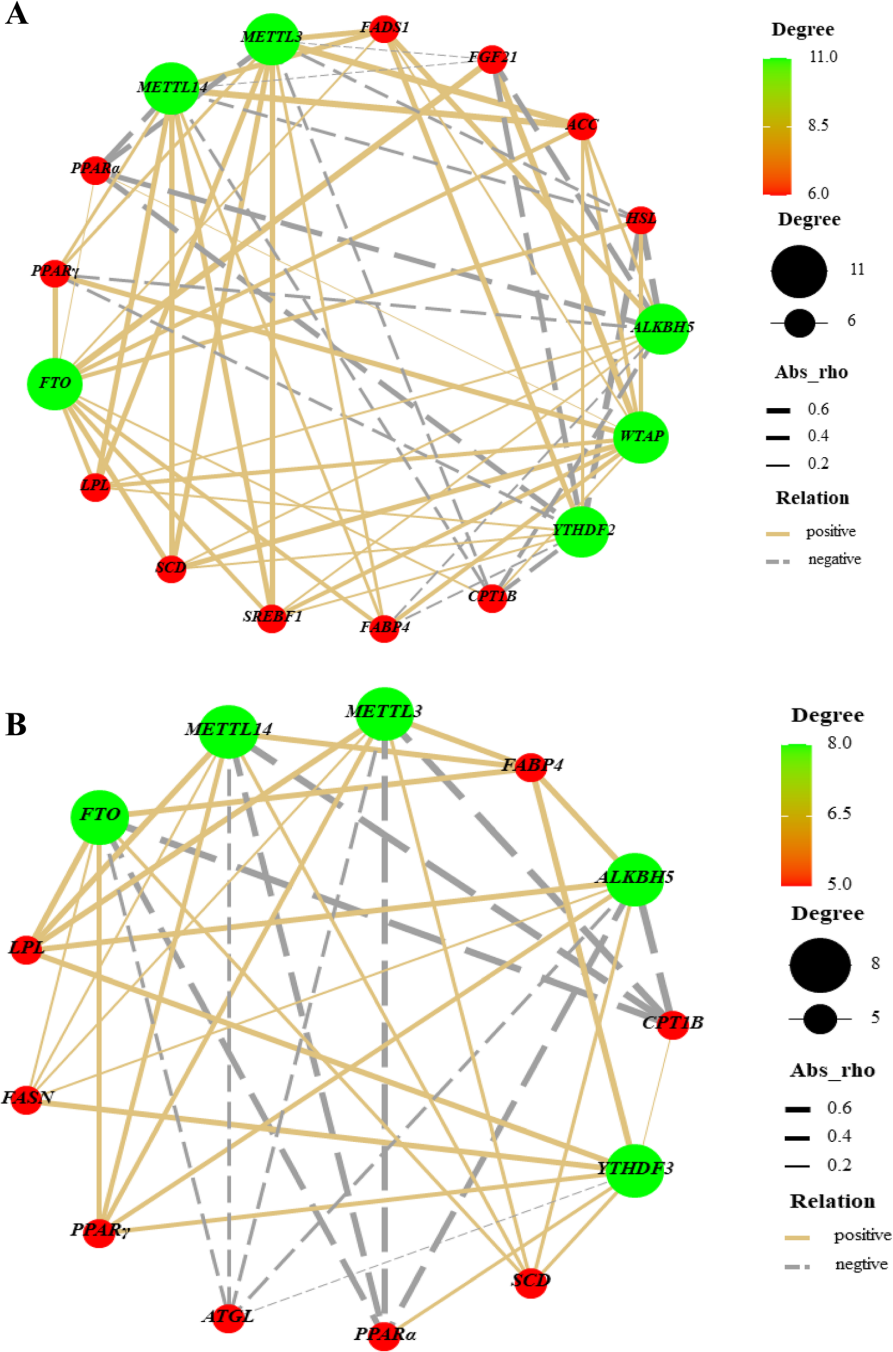
Correlations network analysis among genes related to lipid-metabolism (green circles) and m^6^A methylation (red circles) in the liver (**A**) and muscle (**B**) are presented. Silver lines, negative correlation (*P* ≤ 0.05); Golden lines, positive correlation (*P* ≤ 0.05)

In liver, the mRNA expressions of *ALKBH5* and *YTHDF2* had a negative correlation (*P* < 0.05) with *HSL* and *PPARα* (Fig. 6A). The mRNA expressions of *METTL3* and *METTL14* were positively correlated (*P* < 0.05) with *ACC*, *FADS1*, *SREBF1*, *SCD*, and *LPL* and negatively correlated (*P* < 0.05) with *PPARα*. Likewise, *WTAP* and *FTO* showed a positive association (*P* < 0.05) with *FGF21* and *PPARγ*. In muscle, *YTHDF3* expression was positively correlated (*P* < 0.05) with *LPL*, *FABP4*, and *FASN* (Fig. 6B). Meanwhile, the mRNA expressions of *METTL3* and *METTL14* had a negative association with *CPT1B* and *PPARα* (*P* < 0.05) and a positive association with *FABP4* (*P* < 0.01) and *LPL* (*P* < 0.05). Likewise, *FTO* and *ALKBH5* were negatively correlated (*P* < 0.01) with *CPT1B* and *PPARα* and positively correlated (*P* < 0.01) with *FABP4*, *LPL*, and *PPARγ*.

## Discussion

Following ruminal biohydrogenation, dietary FAs are likely to be further metabolized in the liver and other tissue involved in FAs metabolism despite adipose tissue is a main site of de novo FAs synthesis in sheep [36, 37]. This process could affecting the FAs deposition in the respective tissue. Higher FAs composition in the liver than muscle tissue are not uncommon, and likely reflect the discrepancy of tissues in metabolic roles. In the present study, distinct gene expression and enzyme activity patterns in responses to feeding a LP diet or LP + RML were observed in the liver and muscle tissues. It unveils tissue-specific regulation systems between the inductions of key transcription factor, followed by the activity of genes related to lipogenesis and FAs oxidation. The greater FAs modification in the liver can be accompanied by the greater alters in the expression of *SREBF1*, *ACC*, *FGF21*, and *FADS1* genes responsible for lipogenesis in this tissue. This discrepancy might be attributed to the slight difference in the FAs profiles between the two tissues. In this context, the concentration of C16:0 (palmitic) in the liver of lambs fed a LP diet was increased, which is in line with the greater concentration of plasma TAG and mRNA expression levels of *ACC*, as palmitic is one of the final products of de novo synthesis of SFA. These showed that feeding a LP diet increased the concentration of C16:0 as a result of increased enzymes involved in de novo FA synthesis, thereby increasing the concentration of plasma TAG. In agreement with the present findings, linear patterns between expression of level *ACC* and concentrations of C12:0, C14:0, and C16:0 were reported in cattle under different feeding regimes [38]. In addition, the increased concentration of C16:0 in the liver may be partly due to the high content of C16:0 in the LP and LP + RML groups, which was nearly 5% more than that of the NP diet. Compared with the NP diet group, the relative content of C16:0 in the liver of LP and LP + RML groups was more than 9% and 4%, respectively, which indicates that the addition of RML had an effect on the liver fatty acids synthesis. The observed changed concentration of C15:0 in the present study was likely a result of altered rumen microbial profiles and propionic acid production, which can influence de novo FA synthesis [39].

Feeding a LP diet improved the concentration of C18:1n-9 t in the liver and muscle of the present study and was revered by the supplementation RML with a LP diet, which may be explained by the same expression pattern of *SCD* mRNA. However, the C18:1n-9c concentration did not confirm this pattern. Because the inclusion of RML in the LP diet inhibited the expression of *SCD* mRNA, which plays a significant role in converting C18:0 into C18:1c-9, the concentration of C18:1n-9c did not decrease in this group. Perhaps, there may be additional genes that regulate the conversion of C18:0 to C18:1n-9c. Nevertheless, the increase or decrease in *SCD* activity or expression is not always accompanied by an altering C18:1n-9c or total MUFA concentration. In this context, feeding a LP diet increased the concentration of C18:1n-9 in the adipose tissue without affecting the protein expression of *SCD* [40], suggesting that the concentration of C18:1n-9 increased independent of *SCD* expression in the adipose tissue. Conversely, the inclusion of linseed in a corn-silage-grass hay diet of bulls decreased the mRNA expression of *SCD* in the adipose tissue without affecting the concentration of single C18:1n-9 and total MUFA [41]. The authors stated that feeding a linseed affected the concentration of C18:1n-7 (cis-vaccenic acid). Considering the role of *SCD* gene in the synthesis of conjugated linoleic acid (C18:1c-9 t11) from vaccenic acid (C18:1 t-11) and other single MUFA [42, 43], the increased *SCD* expression in lambs fed a LP diet can also be explained by altering the concentration of other MUFA such as C16:1 and C18:1n-9 t. In addition, a positive association between the expression of *SCD* and concentrations of 14:1, 16:1, and 18:1n-9 were also reported elsewhere [44, 45].

As of C18:2n-6 is the precursor of the n-6 long-chain PUFA series built via extension and desaturation of omega-3/6 FAs [46]. The observed lower concentration of C18:2n-6 and C18:3n-3 in the liver of lambs fed with a LP, or LP + RML diet could be explainable by the relative lower intakes of C-18 unsaturated FA from soybean meal and its ruminal biohydrogenation. Decreased concentrations of PUFA 18:2 and 18:3 in the milk fat were also found in the cows fed a diet containing a reduced level of C-18 unsaturated FA [47]. In addition, the lower concentrations of single PUFA in the liver of lambs fed the LP + RML diet could be partly accompanied by the decreased expression level of *FADS1* in the present study, which is a rate-limiting enzyme in de novo PUFA synthesis [48]. However, the single PUFA in the liver of LP did not confirm this pattern. Other members of the FADS gene family, such as *FADS2*, may be responsible for regulating the synthesis of single PUFA under LP diet conditions. In the present study, the FAs results showed increased concentrations of single SFA and total SFA and decreased total PUFA proportions in the liver, but not in the muscle of lambs fed the LP diet. In concordance with the present results, feeding a LP diet did not affect the single or partial sums of SFA and total SFA and PUFA in the muscle of yearling lamb [49]. The results indicated that reducing dietary protein levels by 2% does not have pronounced effects on the partial sums of SFA, MUFA, and PUFA in the muscle of lambs. However, a significant shift in the FA composition of pigs fed LP diets was reported elsewhere [50], which could be explained by breed differences.

To further confirm whether a LP diet supplemented with or without RML alters the lipid metabolism, we assessed the activity or expressions of the lipogenic and lipolytic enzymes. The malonyl-CoA, believed to be a rate-limiting enzyme in the process of FAs synthesis, is synthesized from acetyl CoA by the enzyme ACC [51]. Then, the synthesized malonyl-CoA is converted into FAs in the presence of FAS enzyme and NADPH as hydride donor [52]. The SCD is a vital and rate-limiting enzyme involved in MUFA synthesis from SFA by lodging a double bond between two carbons of the fatty acyl chain [43]. Supplementation of RML with a LP diet down-regulated the expression of lipogenesis *ACC*, *SCD*, and *FADS1* mRNA in the liver but not in the muscle, suggesting that the inclusion of RML in a LP diet depress lipogenic genes involved in the de novo FAs synthesis via activating transcription factor *SREBF1*. It could also be explainable that supplementation of RML with a LP diet decreased the plasm TAG concentration by regulating genes involved in FAs synthesis.

The *SREBF1* is a key transcriptional factor that regulates genes implicated in the de novo synthesis of FAs and TAG and maintains cellular lipid homeostasis [53]. The lack of changes in the expression of *ACC*, *FADS1*, and *FGF21* in the muscle of the present study are consistent with the expression of *SREBF1*, which may preferentially regulate or activate those genes for the de novo lipogenesis. It could also be explainable by the limited role of *ACC*, *FADS1*, and *FGF21* genes in the lipogenesis-associated pathways of muscle under the present experimental condition. These results were agreed with the previous findings that showed the expression of lipogenic enzymes in the muscle was lower than in liver or adipose tissues of lambs and cattle under different feeding regimes [39, 48]. The transcription factor *SREBF1* has also been shown to mediate the expression of *FGF21* in the hepatocytes [54], which influences lipid accretion and mitochondrial oxidative capacity of the fat cells. Xu et al. [55] have confirmed using goat intramuscular preadipocyte that *FGF21* could be negatively regulated by *SREBP1* during adipogenic differentiation. Higher mRNA expression of *FGF21* has been observed in the liver of mice fed a LP diet [56]. In the present study, the supplementation of LP diet increased the expression of liver *FGF21*, accompanied by increased expression of *SREBF1*, reinforcing the involvement of *FGF21* in the lipid metabolism in the liver of lamb under LP protein diet conditions. However, this change was not observed in the muscle of lambs, suggesting that it is mainly produced in the liver than muscle.

The *FABP4* is a carrier protein responsible for the carting of free FAs, released from extra-or-intracellular hydrolysis of TAG in the presence of *ATGL* and *HSL*, into the specific fat cells. After that, acyl-CoA is carted into mitochondria for β-oxidation following its production from free FAs [57]. Supplementation of RML with a LP diet increased the activity of *HSL* or *ATGL* and decreased the expression of *FAPB4* in the liver and muscle of the present study. It would be explainable that the *HSL* or *ATGL* could undergo the hydrolysis of intracellular TAG to release free FAs, thereby transported by *FAPB4* into the fat cell, which contributes to the alteration of fat deposition. The *LPL* is believed to be responsible for the hydrolysis of extracellular TAG in lipoproteins or TAG-rich lipoproteins and regulates TAG portioning between storage in adipose tissues and oxidation in muscle, thereby supplying appropriate FAs as fuel for muscle growth [58]. The inclusion of RML in a LP diet down-regulated the expression of *LPL* in the liver and muscle of the present study, suggesting that the availability of FAs as an energy source for the respective tissue was reduced. Another significant finding of the present study is that increased mRNA expression of *PPARα* in the liver and muscle of lambs fed a diet consisting of RML, suggesting that the inclusion of RML in a LP diet promotes FAs oxidation via activating *PPARα*. The *PPARα* is a major transcriptional activator or sensor of FA oxidation and plays a crucial role in this process, whereby the synthesis of fatty acids is influenced [59]. Elevated mRNA expression of *CPT1B* in both tissues of the present study was accompanied by an increased expression of *PPARα* or concentration of endogenous carnitine. Perhaps, dietary Lys and Met, an exogenous precursor for L-carnitine biosynthesis, could increase the expression of *CPT1B* that regulated FAs catabolism through either translocating the FA into mitochondria or regulating the enrollment of FAs flux into the β-oxidation pathway [60]. Thus, this study clearly showed that the inclusion of RML in a LP diet could promote lipolysis by regulating the transcriptional factors and downstream genes, which eventually led to low-fat accretion in the respective tissues of lambs.

Studies have been shown the role of m^6^A RNA methylation in regulating fat metabolism in lambs [61] and pigs [17]. Lipid accretions are inversely regulated by m^6^A RNA methylation in pigs [17, 62]. A recent study has shown that methyl donors, such as betaine, can regulate the m^6^A RNA methylation in HepG2 cells [15]. Considering Met and Lys regulate methylation and Met as a methyl donor, we measured the total m^6^A methylation level and the mRNA expressions of methylases and demethylases enzymes to assess the impacts of a LP diet supplemented with and without RML on m^6^A RNA modification. The results showed that supplementation of RML with a LP diet increased the m^6^A RNA methylation in the liver and muscle. The increased m^6^A RNA methylation was accompanied by the increased concentrations of SAM, which is the main methyl donor synthesized from Met through one-carbon metabolism [13].

Furthermore, the observed decreased expressions of FTO and ALKBH5 enzymes in the present study of liver and muscle were accompanied by increased the relative m^6^A levels in the RML supplemented group or decreased expression of transcription factor *PPARγ*. These results reinforced the inverse association of *FTO* and m^6^A [14, 62] or showed the influences of *FTO* in fat metabolism partly through regulating the expression levels of *PPARγ* [63]. In this regard, supplementation of betaine to mice reversed high-fat diet-induced hepatic lipid accretion via down-regulated expression of *FTO*, thereby increasing the level of m^6^A [14]. Wang et al. [62] showed using a methyl donor (betaine) and methylation inhibitor (cycloleucine) that levels of m^6^A could regulate the lipid accretion via *FTO* in porcine adipocytes, showing that FTO-dependent removal of m^6^A has a vital role to regulate fat deposition. In addition, a mechanistic influence of *FTO* on the expression of *PPARγ* during preadipocyte differentiation was reported [63, 64]. These researches indicated that m^6^A could regulate fat deposition directly through *FTO* or indirectly through *PPARγ.* In the present study, the inclusion of RML in a LP diet increased the m^6^A status via decreased expression of *FTO*, and the decreased mRNA expression of *FTO* was consistent with that of *PPARγ* in both tissues, suggesting that m^6^A could involve in lipid metabolism by controlling the expression of *FTO* and (or) *PPARγ* in lambs. These results were further supported by the correlation analysis that showed a positive association between mRNA expressions of *FTO* and *PPARγ* in both tissues.

It has also been documented that m^6^A methylation is positively and negatively associated with methyltransferase *METTL3* and demethylase *FTO* in porcine adipocytes, respectively [62]. A positive association between m^6^A and methyltransferase has been observed in lambs exposed to heat stress [61]. However, the positive association between m^6^A and methyltransferase was not observed in the liver and muscle of the present study. The possible reason might be explained by the increased concentration of SAH, which is reported to have strong inhibitory effects on the activity of *METTL3* [65]. In addition, the mRNA or protein expression levels of METTL and WTAP partly failed to show a positive correlation with m^6^A RNA methylation in the liver and adipose tissue of pigs fed different level of branched-chain AAs [17]. It would also be explained that other m^6^A writers could be responsible for the m^6^A deposition process, which needs further investigation. Recently, several m^6^A writers, including *METTL16* and *PCIFI*, have been identified, which could be a potential regulators [66, 67].

Growing evidence showed that the *YTHDF1–3*, m^6^A-binding proteins, are obliged to recognize the m^6^A modification and promote various downstream effects [16]. The *YTHDF1* has been found to control the expression of mitochondrial carrier 2 protein and trigger mRNA translation [68], while *YTHDF2* relates to admitting m^6^A-modified mRNA and influences its stability [69]. In this study, supplementation of RML with a LP diet down-regulated the expressions of liver *YTHDF2* and muscle *YTHDF3*, suggesting that limited m^6^A-binding proteins recruited the m^6^A-modified mRNA. Other m6A reader proteins that might be involved in the RML-induced prevention of lipid accretion can explain the lack of changes in m^6^A-binding proteins. Overall, the current study highlighted that dietary Met and Lys influenced m^6^A RNA methylation, methyltransferase, and demethylase enzymes, and were more pronounced in the liver than the muscle. The increased m^6^A RNA methylation and decreased expressions of *FTO* and transcription factor *PPARγ* in this study reinforced the potential role of RML in modulating lipid metabolism through modification of m^6^A RNA methylation.

## Conclusions

Our results indicated that feeding a low-protein diet increased lipogenic enzymes and decreased lipolytic enzymes in the liver and muscle compared with a NP diet, which was reversed by the inclusion of methionine and lysine supplementation in a LP diet. The composition of fatty acids was more affected in the liver than in the muscle, which may be linked with higher mRNA expression of the vital lipogenic enzymes and adipogenic transcription factors. Dietary methionine and lysine in a low-protein diet could influence lipid metabolism via m^6^A RNA methylation, as evidenced by the decreased demethylases of *ALKBH5* and *FTO*, which may regulate lipid accretion through *PPARγ*.

## Supplementary Information

**Additional file 1: Table S1. **Primer sequences and amplicon information.

## Data Availability

All data generated or analyzed during this study are available from the corresponding author upon reasonable request.

## Abbreviations

LP: Low-protein
NP: Adequate protein
RML: Rumen-protected methionine and lysine
LBW: Live body weight, kg
ADG: Average daily gain
ADFI: Average daily feed intake
HCW: Hot carcass weight
DP: Dressing percentage
m^6^A: N6-methyl-adenosin
FAs: Fatty acids profiles
SFA: Saturated fatty acids
MUFA: Monounsaturated fatty acids
PUFA: Polyunsaturated fatty acids
FAME: Fatty acid methyl esters
FADS1: Fatty acdis desaturase-1
ACC: Acetyl-CoA carboxylase
FTO: Fat mass and obesity-associated protein
ALKBH5: AlkB homologue 5
METTL3: Methyltransferase-like 3
METTL14: Methyltransferase-like 14
YTHDF1–3: YTH domain family proteins 1–3
TAG: Triglycerides
HDL-C: High-density lipoprotein-cholesterol
TC: Total cholesterol
LDL-C: Low-density lipoprotein-cholesterol
NEFA: Non-esterified fatty acid
LEP: Leptin
ADPN: Adiponectin
INSU: Insulin
FGF21: Fibroblast growth factor 21
HSL: Hormone-sensitive lipase
ME: Malic enzyme
LPL: Lipoprotein lipase
CPT1B: Carnitine palmitoyltransferase I B
FABP4: Fatty acid binding protein 4
FADS1: Fatty acid desaturase 1
SCD: Stearoyl-CoA desaturase
SAH: S-adenosylhomocysteine
ATGL: Adipose triglyceride lipase
FASN: Fatty acid synthase
SREBF-1: Sterol regulatory element-binding protein-1
PPARγ: Peroxisome proliferator-activated receptor-γ
PPARα: Peroxisome proliferator-activated receptor-α
GAPDH: Glyceraldehyde-3-phosphate dehydrogenase
RPS9: Ribosomal protein S9
