# Supplementary material for: Lipid metabolism and m6A RNA methylation are altered in lambs supplemented rumen-protected methionine and lysine in a low-protein diet
Source: J Anim Sci Biotechnol. 2022 Jul 13;13:85. doi: 10.1186/s40104-022-00733-z (PMC9277831; doi:10.1186/s40104-022-00733-z)
Supplement: Supplementary file 1 — Additional file 1: Table S1. Primer sequences and amplicon information. [file 40104_2022_733_MOESM1_ESM.doc]

**Table S1.** Primer sequences and amplicon information

| **Gene** |  | **Primers (5′ to 3′)** | **Bp** | **Accession #** |
| --- | --- | --- | --- | --- |
| *ACC* | Forward | GTGAGCCTGCGGAATA | 238 | NM_001009256.1 |
|  | Reverse | TGCCGTCATAGGACAAGA |  |  |
| *FASN* | Forward | CTCGGTGCCCGTTGTCTA | 188 | XM_027974304.2 |
|  | Reverse | GGAGGTATGCCCGCTTTT |  |  |
| *SCD* | Forward | AGTACCGCTGGCACATCAAC | 100 | NM_001009254.1 |
|  | Reverse | AAGACGGCAGCCTTGGATAC |  |  |
| *HSL* | Forward | ACAGCAGCGACACAACAGAC | 108 | NM_001128154.1 |
|  | Reverse | CAGATTCATCCTCAGACCCAAG |  |  |
| *LPL* | Forward | CCAGCAGCATTATCCAGTGTC | 120 | NM_001009394.1 |
|  | Reverse | CCCAAGAGATGCACATTACCC |  |  |
| *ATGL* | Forward | CATGGAGAGGACTGGAGGGA | 84 | NM_001308576.1 |
|  | Reverse | TCATTGAGCCTTGAGGGCAG |  |  |
| *CPT1B* | Forward | TGTTCAACACCACTCGCATC | 116 | NM_001009259.1 |
|  | Reverse | CTCGTAGAGCCACAGCTTGA |  |  |
| *FADS1* | Forward | TCCGCAAAGACCCTGACATC | 82 | XM_004019593.5 |
|  | Reverse | TTCTGTTTCCCGAGCTCCAC |  |  |
| *FABP4* | Forward | AAACTGGGATGGGAAATCAACC | 109 | NM_001114667.1 |
|  | Reverse | TGCTCTCTCGTAAACTCTGGTAGC |  |  |
| *PPARγ* | Forward | GCATTTCTGCTCCGCACTAC | 120 | NM_001100921.1 |
|  | Reverse | ATACAGGCTCCACTTTGATTGC |  |  |
| *SREBF1* | Forward | CGCTCGTCTTCCTCTGTC | 90 | XM_027974784.2 |
|  | Reverse | TGATGCTGGTGGTGTCG |  |  |
| *FGF21* | Forward | GCCGGACTCCAAAACAGGAT | 79 | XM_027977590.2 |
|  | Reverse | GTGGTTCACGTCCTCAGGTG |  |  |
| *PPARα* | Forward | TCATGGAGCCCAAGTTCGAC | 126 | XM_027968213.2 |
|  | Reverse | GTCCTACGTTTAGGAGGCCG |  |  |
| *METTL3* | Forward | GACTATTGCAGGGCAGAAGC | 152 | XM_004010362.5 |
|  | Reverse | GATCAACATCTGAGGCAGCA |  |  |
| *METTL14* | Forward | CCCCGTTTTTCCGTCTCCTC | 95 | XM_012179421.3 |
|  | Reverse | CGATTCGGGGTAGAGAGGGT |  |  |
| *WTAP* | Forward | CTCCGTTTGGAGAGGATTCAA | 237 | XM_004011435.5 |
|  | Reverse | CTGCGTGCAGATTCTTGCTG |  |  |
| *FTO* | Forward | TCTCGAATTGCCCGGACTTT | 85 | NM_001104931.1 |
|  | Reverse | GCAGAGGCATCGAAGGATCA |  |  |
| *ALKBH5* | Forward | ACTGTGCTCAGTGGATACGC | 136 | XM_004023544.5 |
|  | Reverse | TGCTCAGGGACTTGGTTTCC |  |  |
| *YTHDF1* | Forward | TTCAGCCTACGGGAACAGCTAC | 192 | XM_015099784.3 |
|  | Reverse | CTGACGACCGACCCTACTGTCT |  |  |
| *YTHDF2* | Forward | GAACGTCAAGGTCGTGGGAA | 153 | XM_027965411.2 |
|  | Reverse | ACTGCAAGTCTGCAATCGTC |  |  |
| *YTHDF3* | Forward | TGCGTATGCTGGAGTCTGGT | 287 | XM_027972993.2 |
|  | Reverse | TATTTCTCTCCCTACGCATGGC |  |  |
| *ACTB* | Forward | TCGAGCATCCCCAAAGTTCT | 139 | NM_001009784.3 |
|  | Reverse | AGAAGGAGGGTGGCTTTTGG |  |  |
| *GAPDH* | Forward | GGCGTGAACCACGAGAAGTA | 141 | NM_001190390.1 |
|  | Reverse | GGCGTGGACAGTGGTCATAA |  |  |
| *RPS9* | Forward | GAGGCTCTTGGCGTAGTTGT | 190 | XM_027978859.2 |
|  | Reverse | AGCTTCAGCTCTTGGTCGAG |  |  |

Bp, amplicon size in base pair.

Acetyl-CoA carboxylase α (*ACC*), fatty acid synthase (*FASN*), stearoyl-CoA desaturase (*SCD*), hormone-sensitive lipase (*HSL*), lipoprotein lipase (*LPL*), adipose triglyceride lipase (*ATGL*), carnitine palmitoyltransferase I B (*CPT1B*), Stearoyl-CoA desaturase (*SCD*), fatty acid transport protein 1 (*FATP1*), fatty acid binding protein 4 (*FABP4*), peroxisome proliferator-activated receptor-γ (*PPARγ*), and sterol regulatory element binding protein-1 (*SREBF1*), fibroblast growth factor 21 (*FGF21*), peroxisome proliferator-activated receptor-α (*PPARα*), Fat mass and obesity-associated protein (*FTO*), AlkB homologue 5 (*ALKBH5*), Methyltransferase-like 3 (*METTL3*), Methyltransferase-like 14 (*METTL14*), YTH domain family proteins 1-3 (*YTHDF1-3*), glyceraldehyde-3-phosphate dehydrogenase (*GAPDH*) and ribosomal protein S9 (*RPS9*)
